# Histoire de la Medecine depuis son Origine jusqu'au XIX. Siecle

**Published:** 1847-04-01

**Authors:** 


I. Histoire de la Medecine depuis son Origine jusqu'au XIX. Siecle. Par le Dr. *P. V. Renouard.* Tom. II. pp. 980. 8vo. Paris, 1846.

II. Essai sur l'Histoire et la Philosophie de la Chirurgie. Par M. *Malgaigne.*

[Concluded from page 316.]

*Medical Organization in Europe during the Arabic Period.*—In Europe, amidst the chaotic confusion consequent upon the barbaric invasions, the ecclesiastical schools placed under the protection of the bishops, alone retained a slight image of literary and scientific instruction. The exercise of all the liberal professions, and especially of medicine, thus fell into the hands of the clergy; and we read of priests, abbots, and bishops officiating as the physicians of kings and popes. Several of the female religious orders likewise meddled with the practice of physic. Between the 9th and 13th centuries, the Jews, in spite of the canons of the Church which forbad them, divided this monopoly with the clergy. Several of them learned in Arabic were enabled to peruse the medical works in that language, and acquired a degree of skill in their treatment which caused their services to be sought in the courts of princes and even in the palaces of pontiffs. The acquirements of these practitioners, however, whether Jewish or Christian, were usually of the most meagre character; medical education, by reason of the rarity of books and the want of teachers, being an impossibility. No restriction on, or examination for, practice being in existence, crowds of low ignorant persons, barbers, bathmen, and women assumed the titles of curers of disease. Although the law provided no security against ignorant persons assuming these functions, it visited accidents which resulted from their ministration with fine or imprisonment. M. Malgaigne suggests that such severity was especially employed in surgical cases, the practice in which was abandoned to persons of the lowest condition, internal medicine only being in the hands of the clergy. It is probably from about the 7th century that medicine became disunited from surgery. This separation, so little rational in itself, was in violation of the traditions of the great masters, and was gradually brought about by the ecclesiastical prohibitions against the clergy spilling blood, prohibitions which, from their frequent iteration by popes and councils, accompanied by the severest menaces, seem to have been constantly violated. However, in the course of the 12th century, the secular authority commenced endeavouring to remedy the abuses produced by this pernicious exercise of medicine. Roger of Sicily seems to have been the first sovereign who (1140) published an ordinance compelling those who would practice physic to obtain an authorization, and other monarchs gradually followed his example; while the institution of university grades and faculties completed medical organization.

5. *The School of Salerno.*—The origin of this celebrated school is somewhat obscure. It was said to have been founded by some of the refugees from Alexandria; but the period of its greatest splendour was from the tenth to the thirteenth century. The great reputation its professors obtained, far exceeding that of any other part of Christendom, induced persons afflicted with diseases difficult of cure to repair to it from all parts of Europe. Among the most illustrious of the professors was *Constantinus Africanus,* who flourished in the middle of the eleventh century, and rendered great service to his epoch by his compilations from the Greeks and Arabs, and powerfully contributed to popularize the knowledge of the East, in an age remarkable for its ignorance. During the thirteenth century, Frederick II. of Naples decreed that no one should practise medicine without having first been received into the College of Salerno—the course of instruction occupying five years according to Sprengel, and but two according to Malgaigne. The candidate was examined in the therapeutics of Galen, the first book of Avicenna, and the Aphorisms of Hippocrates, and compelled to take an oath that he would exercise his profession honourably, and not participate in the gains of the apothecaries. He who desired to exclusively practise surgery, was obliged to study only for a year, especially directing his attention to anatomy. The celebrated Dietetic Precepts of the School of Salerno, composed in 1100 for the use of Robert Duke of Normandy, who repaired there for the cure of his wounds on his return from the Crusades, has long excited much attention and been the subject of numerous commentaries.

6. *Origin and Development of Universities.*—" In the time of Charlemagne, every Cathedral possessed its school, and in some of these the elements of medicine were taught. When the medical profession had been declared by many councils incompatible with the priesthood, the Popes, in order to retain the jurisdiction they had so long maintained over the members of the medical body and the bar, erected certain of these schools into *Universities,* in which were taught philosophy, theology, laws, and medicine, or only some of these faculties. In this way arose, during the thirteenth century, most of the principal European universities. 'All science,' says M. Malgaigne (Introd. to the works of Ambrose Paré), was in the hands of clerks, and instruction for having quitted the cloister was no less catholic. These new clerks, attached to the head of the church by their oaths and their privileges, constituted for him a numerous and powerful militia; and while, by the clergy properly so called, the popes reigned over the consciences, by means of the university clerks they governed the intellect. Who can feel surprised that they should impatiently desire to concentrate all their power in the same hands?' We must, however, render the act of justice to the popes, monks, and clergy of allowing that they prepared the way for the intellectual movements of modern Europe. The universities, by bringing studious men together, afforded them the occasion and the means of mutual enlightenment, excited their emulation by honours and rewards, and, in fine, contributed very efficaciously to the elevation of the Christian civilization above all others.

" The great effects of these liberal institutions, it is true, were not immediately perceived. It required several generations to develop the consequences and ripen the fruits. This is why the end of this historical period, less barbarous than its commencement though it be, has transmitted to us few names worthy to arrest our attention. Men who then gained a reputation in science, and especially in medicine, shone less by reason of the merits of their works, than by their love for instruction, and the zeal they exhibited in seeking this and propagating it. At the present day, when literary riches abound, we can with difficulty form an idea of the price they must have cost our ancestors. We are astonished at beholding them making journies as expensive as painful: without any encouragement or hope of remuneration, merely to obtain a manuscript or hear a renowned professor." P. 450.

Among the learned persons enumerated by M. Renouard who, at this period, made some efforts to restore the fallen condition of medical science, we can only briefly notice the most famous one of them, *Guy de Chauliac*, who flourished at the commencement of the 14th century. He studied medicine at various schools, was deeply versed in the ancient authors, and became himself a prolific writer, exhibiting, as M. Malgaigne observes, a certain independence in form and criticism not found among the Arabic and Greek commentators. His *Magna Chirurgia*, which he terms the *Inventory* is the principal work which he produced. In it he exhibits a vast erudition, and thus happily reconciles the services of the ancients and moderns. " The sciences are formed by successive additions, and the same men cannot lay their foundation and conduct them to perfection. We are as children riding on the neck of a giant; aided by the works of our predecessors, we are enabled to see all that they saw, and something more." He traces the requisites of a surgeon, M. Malgaigne adds, with a nobleness of language and terseness of expression which the medical world had not seen since the time of Hippocrates. He says, " he should be well-read, expert, ingenious, and very morigenous," that is, according to his own interpretation of this last word, bold in sure things, careful in danger; he should avoid bad cures and practices; he should be kind to his patients, indulgent to his colleagues, and wise in his predictions; he should be chaste, sober, compassionate, and merciful, not greedy or extortionate for money; receiving a moderate recompense according to his labours, his dignity, the circumstances of his patient, and the nature of the issue or event." He insists upon the necessity of dissections, observing that, for some time, anatomical demonstrations had been made on animals at Montpellier, and suggests the expediency of their also being performed on the bodies of criminals. He is the first author who alludes to some anatomical plates, which were designed by one Mandeville. His work, giving a critical abstract of the surgical writings of all prior authors, and that at a period when books were both so scarce and so dear, did immense service for the surgeons of the epoch. For nearly two centuries it became " the surgical code of Europe; translated and commented upon in all languages, and reproduced in various forms, it has long been a classic, and preserves some of its interest even at the present day, as exhibiting the state of the science at the end of the Middle Ages." Guy punctured the abdomen in ascites, attempted the radical cure of hernia, and seems to have operated for cataract; but, although he describes lithotomy, after the Arabs, he left its performance to the itinerant adventurers who at that period undertook it.

7. *Accessary Institutions.*—Charitable establishments multiplied wonderfully during this period, both among the Mahomedans and the Christians; and numerous religious associations devoted their attention to the sick. Sovereigns and Popes not unfrequently offered examples by dressing leprous sores with their own hands: and at no period of history were zealous exertions more required. The leprosy and other cutaneous diseases favoured by the misery and filthy habits of the period, spread with frightful rapidity; and, at the end of the 13th century, at least 2000 leper-houses existed in France alone, and 19,000 in entire Europe. The severe precautions the lepers were surrounded by, and the cruel privations they were submitted to, testify the consternation the disease excited, and the noble devotion of those who resigned themselves to taking charge of these unfortunate outcasts.

An institution, the establishment of *public baths*, which became general in most cities, doubtless contributed far more than any coercive measures to the diminution of cutaneous disease. In the 15th century, the bath-men had formed a powerful brotherhood in Paris; and Despars, physician of Charles VI., and one of the most renowned professors of the faculty, having too openly protested against some abuses the baths led to, was compelled, by the persecutions this company raised against him, to quit the capital.

### III. The Age of Renovation.

[Extending from the commencement of the 15th Century to the Present Time.]

### 7. *The Erudite Period.*

[Comprehending the 15th and 16th Centuries.]

M. Renouard characterizes this epoch *Erudite*, on account of the renewed attention which now began to be paid to the ancient medical classics. Europe had long been content to receive them with all the imperfections of the Arabic versions; but, during the 15th century, a taste for Greek literature sprang up, which was immensely reinforced by the dispersion of numbers of learned Greeks upon the taking of Constantinople by the Turks in 1483. They for the most part fled to Italy, where they were well received and patronised by the Roman Pontiffs, the Medici and other potentates. From this period, translating, collating, and commenting upon the Greek authors became the favourite occupation of the learned, and the task was executed with unwearied diligence, remarkable care, and great sagacity, men of the very highest talent and attainments frequently devoting themselves to its accomplishment. From among the learned physicians who were especially instrumental in diffusing this knowledge of the Greek writers, the author cites the names of *Nicholas Leonicenus* and *Thomas Linacre*, both of whom repaired to Italy in order to instruct themselves from the lips of the refugee Greeks. Leonicenus constantly inveighs against the predilection which his cotemporaries manifested for the Arabic writers, and translated from Greek into Latin for their use, the Aphorisms of Hippocrates and several of Galen's books. *Linacre* also, upon his return to England from Italy, published some excellent translations from Galen, and became indeed the prime mover in reviving the study of the classic languages in this country. He enjoyed great consideration, founded chairs at Oxford and Cambridge, and, in 1518, laid the foundation of the earliest institution—the College of Physicians—which this country possessed for the examination of the competency of persons desirous of practising physic.

The following is an abstract of the views of the progress of the several branches of medical science furnished by M. Renouard.

1. *Anatomy and Physiology.*—For a long period, pigs, monkies, and other of the lower animals were alone submitted to anatomical inspection, and the dissection of the human bodies by *Mondini* at Bologna, in 1315, was an act of singular boldness. Shortly after, he published a small manual of anatomy, illustrated by wood-cuts, and although the descriptions it contained were very imperfect, it constituted the only text-book in the schools, besides the writings of Galen and the Arabists, for more than two centuries afterwards. Towards the end of the 15th and beginning of the 16th century, the prejudices against anatomy began, however, to diminish, the Popes removing their interdictions against it, and the Italian universities furnishing the first opportunities for its public pursuit. Dissections were performed at Bologna, Padua, and Pavia before 1500, and shortly after this the celebrated *Sylvius* pursued the study of anatomy with great vigour at Paris; none of these admitting anything contrary to the authority of Galen, but as a derogation from the ordinary laws of Nature, or as a result of the degeneracy of the human race. At last, however, a true man of genius, *Andrew Vesalius* appeared. A hard student in ancient literature, he also early devoted his attention to anatomy, dissecting while yet a boy every animal he could meet with, and stealing his first skeleton from the gibbet itself. Arrived in Paris, he was not contented with the lectures of Sylvius, but still sought every opportunity for examining Nature, even to the extent of disinterring bodies in the cemeteries—a practice at that superstitious period attended with risk to life itself. So rapid was his progress that, at 23, he was nominated professor of anatomy at Padua, and at 29, published his famous work (*De Humani Corporis Fabricâ*, 1543). The following year he was called to the Court of Madrid as physician to Charles V. and abandoned his anatomical studies, never again to resume them. Vesalius was the first who ventured to expose the errors of Galen, arising, as he said, from the fact of his observations having been made on apes. This raised a violent opposition to his researches, headed by his old master Sylvius: but, as the young Professor founded his criticism upon the observation of Nature, he eventually triumphed. The blow once struck against the predominance of Galen, was amply followed up by the labours of *Columbus, Eustachius* and *Fallopius*, cotemporaries and friends of Vesalius, and who must share with him the honour of laying the foundation of *Modern Anatomy*. Dissecting-rooms were now opened, and unclaimed bodies as well as those of criminals were given up to the anatomist. The Pontiffs at Rome, who had so long obstructed the progress of anatomy, now took the initiative in forwarding it; and thus we find that *Eustachius* of that city had an immense number of bodies at his disposal, while Vesalius could only obtain two or three in a year at Padua. Anatomical plates represented the parts dissected with great accuracy; and the anatomical discoveries made were numerous. The true structure of the heart was now first demonstrated, and the recognition of the valves of the blood-vessels gave the first inkling of the discovery, which was presently to immortalize Harvey.

2. *Hygiene* was much neglected during the disturbed condition and the prevalence of extreme religious observances of the Middle Ages; and for several centuries the dietetic code of the *School of Salerno* enjoyed a reputation which its intrinsic merits in no-wise justifies. The earlier works upon the subject, after the Revival of Letters, were mere compilations from the ancients, and the first we have at all of an original stamp, is the well-known treatise on diet by *Louis Cornaro*, which, according to Hallé and Nysten, has done much to fix the principles and advance the progress of the art. The work of *Mercurialis* also upon the *Gymnastics of the Ancients*, although containing no novel doctrines, was of service in awakening attention to observances long abandoned and forgotten.

3. *Internal Pathology.*—The period in which we are now engaged is chiefly remarkable for its reproduction of the ancient authors, and preparing the way for modern discoveries. Upon *Semeiology*, by far the most elaborate writer was *Fernel.* He minutely considers the various symptoms—especially the pulse and urine—not in the synthetical manner advocated by Hippocrates, but in the analytical mode of Galen. About the end of this period, a new branch of pathology, destined hereafter to acquire so much importance under the title of *Pathological Anatomy*, began to be developed. Benirieni of Florence laid the first foundations of its study at the end of the 15th century, Eustachius and other anatomists following in his footsteps, but not in a sufficiently continuous manner to lead as yet to the establishment of any doctrine. *Nosography.* Of all the treatises of pathology published during this period, that of *Fernel*, attained the most durable and universal reputation, and consisting as it did of a most lucid and complete compendium of the Galeno-Arabic doctrines, it long formed the class-book of entire Europe. On this account, M. Renouard devotes much space to the consideration of his nosological arrangement; but so defective was this, that we need not follow his example. A servile follower of Galen, he furnishes far less accurate pictures of the various species of disease than did Aræteus, Cœlius, or Alexander of Tralles. Towards the end of the 16th century, a distinguished Swiss, *Felix Plater*, opened up a new æra in Nosography, by attempting a classification of disease, founded upon their sensible characters instead of their supposed essential nature.

4. *Therapeutics.*—As this period was famous rather for a revival of ancient methods of cure than the introduction of new ones, M. Renouard deems it a fitting opportunity to take a parting glance at some of the former as ably developed in Fernel's work. This author much insists upon the validity of the rule of treating disease by its *contraries*, or, as it is the fashion now to term it, by the *allopathic* method. M. Renouard occupies several pages in demonstrating the fact, which would seem to be obvious enough, that remedies have not been selected because they differ from or resemble in their nature the producing cause of the disease—differences and resemblances which are purely conjectural and founded upon the most superficial and fanciful observation, but because accident, experience, or analogy has taught us their virtues. The terms homœopathy, antipathy, and allopathy are therefore expressions indicative of a knowledge of the properties of remedies and the nature of disease which neither is or ever can be attained, and their employment should be rejected by all enlightened practitioners as foolish and deceptive. A second precept of Fernel's, respecting the *Expectant* mode of treating disease, is worthy of approbation, as circumscribing this within its just limits. He recommends us, when a disease is obscure in its characteristics and not very urgent in its indications, not to be too hasty in resorting to medicines, but to trust more to Nature and regimen, by the instrumentality of which its true character will often be revealed or even its cure effected. A groping medication is usually injurious, and if your patient compels you to do something, at least avoid by your circumspection doing mischief. A third precept, Fernel considers the capital one, namely, that we must seek for and destroy the cause or causes of a disease before attacking the disease itself, which otherwise is liable to constant reproduction. Many affections are not dependent moreover upon a single cause but upon a series of causes, each of which must be destroyed in the order of its generation. As most of these causes were of a hypothetical character, and endlessly subdivided, the attacking them thus in succession gave rise to the most complex and fanciful treatment.

Fernel recognized but three modes of medication. The Evacuating, the Purgative, and the Alterative. By the first, excess of the humours is disposed of, by the second these are purified, and by the third, parts whose action had become vitiated are restored to their normal condition. He endeavoured to diminish the number of articles then overloading the Materia Medica; and makes no mention of the newly-introduced metals, mercury, antimony, gold, and copper. These substances were, in fact, then in the hands of quacks and charlatans, and prescribed with so little discrimination as to produce oftentimes effects as disastrous as the diseases they were employed to remove. It is not surprising, therefore, that a judicious writer, of the highest authority as a medical teacher, should have felt the necessity of caution.

5. *Surgery.*—The progress of Surgery has been usually in advance of that of internal pathology, in consequence of the greater ease with which external diseases may be observed and treated. During the Middle Ages, however, it fell into a yet lower position than did medicine; for, while the latter was in the hands of the only educated portion of the community, the priests, it was practised by barbers, bath-men and the refuse of society. Sprengel states that, in Germany, for a lad to become apprenticed in trade, it was necessary for him to prove that his family did not contain barbers, shepherds, bath-men, or skinners—classes who, in that country alone, furnished the surgeons. The rest of Europe was in no better condition, if we except some few doctors in Italy and Guy de Chauliac in France, who imparted a temporary brilliancy to the art. In explanation of this utter neglect of an art so obviously and especially useful, at a period when wars and combats were so frequent, we must remember that the Church interdicted its priests from its practice, in consequence of which it was followed as a purely mechanical employment by various ignorant members of Society. Most of these operators were perambulatory, and each confined himself to some particular class of operations, as cataract, lithotomy, hernia, &c., often operating by secret processes, which were transmitted as an inheritance from father to son. The elevation of the profession only commenced after the dissipation of the prejudices against dissections; but its progress was very slow until the 16th century, when most of the great anatomists of that period, as Berenger, Vesalius, Fallopius, Fabricius, &c. were also distinguished surgeons; and other distinguished men also joined a profession now become honorable, but no name is so distinguished at the present epoch as that of *Ambrose Paré.*

Paré, born of poor parents, and apprenticed to a country barber-surgeon, was brought, about the year 1532, to Paris by the great desire he felt to qualify himself for the more important duties of the profession. He studied with such earnestness during three years at the Hôtel Dieu as to attract the attention and approbation of its surgeons. In 1536, he joined the army as a surgeon; and relates in his account of the campaign that, being unprovided with the boiling oil, then thought requisite for cauterizing the wounds, he could not sleep for anxiety, and his extreme surprize at finding that the patients deprived of it had suffered less than the others. The doctrines of the day taught that, wounds from fire-arms were of a poisonous character, only to be treated by cauterizing them with boiling oil or the red-hot-iron, and the internal administration of alexipharmics. "Chance put him in the way," says M. Malgaigne, "of making his first discovery; but what was not chance was that quickness and depth of judgment, that boldness of resolution, which immediately induced him—a youth without name or influence, and yet more without any literary or philosophical education—to single out and combat a doctrine universally recognized and maintained by the highest surgical authorities of the period." His reputation rapidly increased, and after the campaign of 1543, Sylvius, whose lectures attracted crowded auditories, expressed a wish to see him, and listened with deep attention to his opinions. He entreated him to publish them, and in 1545, appeared his little work on gun-shot wounds, which amply testified that a new epoch had arrived in French Surgery. In 1552, he performed the signal service to humanity of substituting ligature of the vessels after amputation for their cauterization.

He was made surgeon to several successive sovereigns, but, amidst the noise of camps, a large practice, and multiplied occupations, he found time to read all that was published concerning his art, and to compose himself a great number of works. "He enriched almost every branch of surgery with some discovery or improvement, and, so far from imitating the secresy so much in vogue at the period, he freely communicated all he knew, and indeed felt it a matter of conscience to make it public."

6. *Obstetrics* were studied by a few of the principal surgeons of this period, but by none so specially as by *Guillemeau,* pupil and friend of Paré. To him are due the earliest of modern improvements, among which may be especially mentioned the precept of terminating the labour artificially in the cases of great *hæmorrhage* and *convulsions.* Although the *Cæsarean operation* had been known from the remotest periods, it was only employed in ancient times for the removal of a fœtus from a woman recently dead. A law of Numa Pompilius ordains (as indeed does the Catholic Church at the present day) the opening of the belly of every woman dying with a child in her womb. The earliest authentic example of the operation having been performed on the living woman is not of later date than the fifteenth century.

7. *Clinical Instruction.*—Nothing corresponding to the idea we attach to this term was in force during the Middle Ages. In place of narratives, formed upon the plan of Hippocrates' admirable "Epidemics," we find nothing but sterile disputations upon the principle of life, the essential character and latent causes of disease, &c. Upon the Revival of Letters, even at the period we are now engaged in, the studies were more philological than practical, and that admirable spirit of investigation manifested in some of the ancient writings, now again brought to light, was yet some time in finding imitators. Eventually, however, it did so, as stated in the following extract:

"Nothing proves better how much progress the art of observing and describing pathological phenomena had made progress, than the great number of *new diseases* mentioned by the authors of this period. We read in their writings for the first time of whooping-cough, sweating sickness, scorbutus, plica, raphania, and syphilis. Can we believe that all these affections, some of which so deeply modify the economy, made their irruption upon Europe at the same time? Is it probable that the changes wrought in the commercial and political relations of nations, the discovery of the New World, long sea-voyages now become so frequent—in a word, the modifications introduced into the public and private hygienic observances by so many events characterising the epoch—may have given sudden rise to this deluge of new evils? No one, I think, would venture to maintain this. It is more probable, I would almost say it is certain, that the majority of these diseases were of sufficiently old existence; but that there did not before exist observers sufficiently attentive to discern their proper characters or historians exact enough to describe them.

"The physicians of our own times are divided in opinion only concerning one of these, *Syphilis.* Some incline to the belief that it was spontaneously developed in Europe towards the end of the 15th century; others, that it was imported from the New World; while the greater number regard it but as a *degeneration of one of the numerous modifications of lepra or elephantiasis* which were so prevalent in the Middle Ages."—*Tom.* II. p. 90.

M. Renouard examines these opinions at considerable length, and expresses his belief in the accuracy of the last-named.

8. *Theories and Systems.*—A mixture of Galenism and Arabism formed the dominant theory of the University Schools of entire Europe. The most distinguished teachers, with some few exceptions, employed their entire sagacity, in reconciling the ancient doctrines—those professed by Plato and Aristotle, Hippocrates and Galen, Rhazes and Avicenna. Of these, *John Fernel,* surnamed the "modern Galen," as we have already mentioned, attained a vast influence among his contemporaries, his compilation from the ancients retaining its authority in the Schools long after his decease.

9. *The Occult Sciences.*—Amidst the general prevalence of the Galeno-Arabic doctrines in the schools of Europe, certain voices might be heard protesting against their reception. These had little influence, having to propose, in lieu of doctrines which had received the sanction of ages, only the crudest essays and most fantastic lucubrations. The partisans of the *Occult Sciences* were among those who most resisted the yoke of authority.

"Full of confidence in their own powers, they would only receive the law from themselves. Some of them were not deficient in sagacity, imagination, or boldness; but the majority wanted connection in their ideas, propriety in their language, and dignity in their conduct. Prophets or demons, they had among themselves no community of principles, but lived generally isolated from each other and from the rest of the world, rendering themselves remarkable only by their eccentricities, their contrarieties, and even their misfortunes. Instead of duly guiding the car of reason, these sectarians, who first gave the signal of revolt against received opinions, would have induced its yet farther deviation had the world followed their foolish direction. Nevertheless, we find in their writings some useful truths amidst a load of trashy reveries. They founded no doctrine worthy the attention of philosophers, but, by their declamations, they forced the true *savant* to quit the narrow path of the past, to review the principles of our knowledge, from which revision a scientific reform sprung up during the ensuing period."—*Tom.* II. p. 111.

The first founder of the Occult Sciences mentioned in history is *Cornelius Agrippa*, a man of great and varied acquirements, but possessed of a restless temperament and caustic humour, which prevented his following a fixed and regular life. Sometimes the physician of princes, and at others a vagabond in the face of Europe, or the inhabitant of some of its goals, he composed a curious and extravagant work upon the Uncertainty and Vanity of the Sciences. M. Renouard, after alluding to some of the extravagancies it contains, observes :—

"Errors of science, superstitious prejudices, religious exaltation, and the thirst for riches, concurred at the same period to propagate the follies of the cabal; and never were there seen such numbers of sorcerers, possessed, astrologers, and alchemists, never were prophesies, visions and prodigies of all kinds so common. No remarkable event could occur but immediately it was pretended that it had been announced by this or that precursory sign. How frequently the expectation of the end of the world predicted for a period near at hand, threw entire nations into consternation, and carried anxiety and terror equally to the bosom of the palace as to that of the cottage! In no country were these cabalistic reveries so universally adopted as in Germany, where mysticism maintained them much longer than elsewhere. Luther himself partook of these vulgar prejudices and superstitions, and contributed much to their propagation. He frequently speaks of his struggles with the devil; and relates how the wicked spirit would appear to him as a monk and tempt him with captious syllogisms. He blames physicians for attributing to natural causes a variety of evils of which the devil is alone the author.

"The history of the period everywhere presents the spectacle of the reign of darkness struggling with nearly equal power and success against that of light and truth."—*Tom.* II. p. 118.

*Jerome Cardan* was another of these eccentric beings possessed of vast abilities, but lost to the world for want of due regulation and guidance. "His immense acquisitions," says M. Dezeimeris, "his extraordinary sagacity, his great freedom of thought, and his masculine and elevated style, would have placed him at the head of the most justly celebrated writers of the 16th century, had he not united to these qualities so confirmed a taste for paradox and the marvellous, a childish credulity, a scarcely conceivable superstition, an unsupportable vanity and unlimited boasting." But of all the professors of occult science, the most famous was indisputably

*Paracelsus.*—Still, famous as he was in his day, we think M. Renouard has occupied too large a space in refuting his errors and absurdities. Certainly he may find good excuse for this in opposing the views of an observer generally so accurate as M. Malgaigne, but, who in regard to Paracelsus, captivated by some of his expressions in approval of the exercise of observation, (which, however, he never himself put in force), sees much cause of admiration that even a superficial examination of his writings on medicine must at once dispel. Indeed, the only ground for notice Paracelsus can claim, is his popularizing the employment of various mineral preparations, now among the most powerful agents of our materia medica. But, whatever good may have eventually resulted from this, it is impossible but that the indiscriminate and careless use of these potent instruments in the hands of himself and successors, must have led to much and dire mischief. To this topic we shall return in our notice of Gui Patin's letters, in which it forms a frequent theme. In the mean time, we may conclude this part of our subject with a quotation borrowed by M. Renouard from Sprengel.

"A revolution which has Mysticism for its basis will find much easier access among the common race of men than one founded on good sense, because the chimera of the imagination are always presented in the most vivid colours, and excite the mind much more than the severe deductions of cool reasoning. In the 16th century, Germany enlightened entire Europe by her reforming spirit. The great Genius of Luther rendered to his cotemporaries and to posterity the inappreciable service of striking a death-blow to the mysticism of catholicism and the scholastic philosophy. Paracelsus adopted the same plan; but the following circumstances prevented his system being received with the same favourable and general reception as that of the theological reformer.

"1. Medicine is a science of experience which must be learned to be known. It reposes on reasoning and the deductions of experience, and consequently any doctrine which rejects the testimony of reasoning and represents experience as useless, can never meet with much success among physicians. 2. The system of Paracelsus was not only based upon mysticism, but upon the grossest fanaticism. In fact, superstition reigned tyrannically throughout the course of the 16th century; but, to endeavour to give these very prejudices the appearance of scientific doctrine, was an idea too revolting to common sense to be generally adopted. 3. Lastly, Paracelsus was not the man to secure success for his system. He was a barbarian, an ignoramus, who despised every science, just because he was totally unacquainted with any. Although he spoke much of the divine light the source of all knowledge, his manners and vagabond life furnished no proof of his participating in it.

"Nevertheless, his doctrine found, and especially in Germany, more partisans than would have been supposed. According to my calculation, three-fourths of his successors were Germans, but most of them were persons entirely destitute of education, and ignorant of science, who cast themselves into the midst of his mystical system just because it seemed to conceal their want of instruction and inaptitude. Others employed the medicaments and arcana of Paracelsus, endeavouring to reconcile his theory with the system of Galen; while, lastly, the Rosicrucians applied it in a much more precise manner than had hitherto been done to theology and philosophy."—*History of Med.* Chap. III.

8. *The Reformatory Period.*

[Comprehending the 17th and the 18th Centuries.]

"We have just seen the systems of Aristotle and Galen resist the premature attacks of the partisans of the Occult Sciences, and enlist the majority of intellects, save some partial modifications impressed upon them by less bold and more sensible innovators. The long duration of these systems, the almost unanimous agreement of the great men of antiquity and of the middle ages in their favour, formed them into the most respectable of precedents, which as yet was disdained but by few. It is no-wise astonishing, then, that the most eminent men of science should prefer them to the crotchetty and ill-elaborated theories of the contrivers of the occult doctrines—those restless, capricious, and haughty spirits who attempted to arrogate to themselves the sceptre of knowledge, without having taken any pains to ripen a plan of scientific reform, whose wisdom and grandeur might justify so high a pretension in the eyes of enlightened men.

"Nevertheless, the domain of the natural sciences became enlarged from day to day. During the two last centuries, observation had enriched it with a multitude of new facts which ill squared, or squared not at all, with accredited doctrines; and the time approached when the necessity of a radical reform was felt in almost every branch of human knowledge. Men, whose learning equalled their genius, are about to appear to direct this intellectual movement, and to substitute, for the decrepid theories of the schools, newer and more powerful ones, better harmonizing with the range of ascertained phenomena. To the worship of the ancients is about to succeed an immoderate desire of shaking the yoke of their authority, to as it were avenge their prolonged tyranny. This is why I have termed the period *Reformatory*—a term which, if I mistake not, perfectly characterizes the general tendency of mind, the predominant idea, and the prominent fact of the epoch." *Tom.* II. p. 156.

At the commencement of this period *Galileo* opened the way for the regeneration of physical science, and following the rigid spirit of observation, in place of the fashionable subtleties of the day, he became the parent of several discoveries, any one of which would have served to immortalize him. "The bold and happy hypotheses of *Kepler* also opened up the way of the heavens to Newton." Naturalists now freely availed themselves of the aid afforded by the microscope; while a new race of chemists, abandoning the dreams of alchemy, and adopting experiment as their guiding star, were among the most potent adversaries of the old philosophy. Theories in medicine were now no longer received on account of the ingenuity of their conception, and not only did Galenism, but several new systems as well, receive their death-blow; for, "although these last had been devised and sustained by men of superior merit, they all erred in considering the phenomena of the animal economy but only under some of its points of view, neglecting others just as important. All, too, erred in the no less grave error of exceeding in their abstractions the limits of sensible phenomena. This is why they all vanished, or became entirely modified, after enjoying a more or less ephemeral reputation."

M. Renouard, as in the former periods of this history, but at much greater length, reviews the state of medical science at this epoch, casting a retrospective glance from time to time at the steps by which it has arrived at so improved a condition. We can only sparingly follow him, as we are desirous of alluding, also, to the various novel theories which so remarkably characterized this period of intellectual activity and learned research.

1. *Anatomy and Physiology.*

The anatomists of the 16th century had so accurately described all the most obvious parts of the frame, that little remained for their successors to accomplish in this respect; but, directing their attention to minute and comparative anatomy and to experimental physiology, they reaped some most excellent discoveries.

*The Circulation.*—According to the ancient doctrines, the liver was the organ of sanguification, and there the veins, they being the only vessels which contained blood, took their origin. The blood was supposed to be sent to and return from all parts in these same canals, by a sort of undulatory movement. The arteries were stated to contain the vital spirits, whose great reservoir was the heart. Galen modified this doctrine by shewing that the arteries likewise contained blood. He was aware, too, that this fluid was brought by the large veins into the right cavities of the heart; but he maintained that, while a small portion passed to the lungs by the pulmonary artery, the great mass penetrated the pores of the septum into the left ventricle. This opinion was uncontested until the time of *Servetus* in the 16th century, who denied the passage of the blood through the septum, and maintained that it passed through the lungs by the pulmonary artery, and was returned to the left ventricle by the pulmonary veins. *Columbus* soon after proved this to be correct by anatomy, and explained the true use of the cardiac valves. *Cæsalpinus* added that the minute arterial ramifications communicated with the veins in the lungs. The existence of valves in the veins was already known; so that it seemed to require, at the commencement of the 17th century, but one step for the discovery of the circulation—a difficult one however. *Harvey* attended the lectures of the celebrated Fabricius de Aquapendente at Padua during four years. The venous and cardiac valves were demonstrated by the unconscious professor to his young pupil, thus casting a germ into his fertile mind which years after was to spring up and flourish into the goodliest tree of science. His first announcement of his discovery was in his lectures at the College of Physicians in 1615; but not until 1628 did he consider his observations sufficiently mature for publication. "England," says Haller, "had contributed nothing to anatomical science, when, behold, a man appears, whose discoveries constitute the only grand epoch in its history since the days of Hippocrates." M. Renouard does justice to the admirable course which Harvey adopted, both in pursuing and confirming his investigations, and in meeting the vehement opposition he triumphantly contended with. We are loath to mar so important a theme by a mere incidental allusion; but certain we are that no greater service can be done our profession, and especially the younger members of it, than the taking some more fitting opportunity for exhibiting in detail the lessons deducible from the example of this truly great man.

"This theory, which now appears to us so natural," says M. Renouard, "that we can hardly understand why it was not discovered long before, was however nothing less than a revolution in physiology." Truly so; and we may compendiously say, that a principal occupation of subsequent physiologists and physicians has been to multiply proofs of the truth of the doctrine, explain the mechanism of the various structures concerned, and direct its practical applications to the prevention or removal of disease and the relief of accidents. *Malpighi*, with the aid of the microscope, demonstrated the actual progress of the globules in the small vessels; and *Leuwenhoek* traced the same, before numerous witnesses, into the minutest ramifications. In 1749, *Senac* published his great work on the Heart and its Diseases, which excited so much the admiration of his cotemporaries, and especially of Morgagni. His ideas upon the cause of the movements of the heart were fanciful in the extreme; but the diagnosis of its diseases received all the perfection it was capable of, prior to the discovery of auscultation and percussion.

2. *Respiration.*—Prior to the 17th century, it was believed that the air which penetrated into the bronchial ramifications was in part subtilized and conveyed by the pulmonary veins to the heart for the fabrication of the vital spirits, and in part exhaled with the fuliginous matters of that organ during expiration. In this way, the lungs were cooled and freed from the danger of their proximity to the heart, the centre of animal heat, and an æther or pneuma provided as a source of the vital spirits. The discovery of the circulation destroyed the basis of this theory; and, in 1661, Malpighi demonstrated the cellular structure of the lungs. Borelli, Helvetius, Haller, and others, made numerous experiments upon the manner in which the inspiratory and expiratory movements of the chest are affected, and established the facts as they are now received. Various pneumatic theories were invented, but were unable to hold their ground after the discovery of the changes which the air underwent during respiration. Mayow's experiments in 1668 opened the way for future observers, and *Lavoisier*, towards the end of the 18th century, presented us with his chemical theory, so seductive by its simplicity. With this of course our readers are familiar, as with the objection derivable from the influence of the nervous system not having been taken into account. M. Renouard takes no notice of Dr. Adair Crawford's important Essay, so confirmatory of *Lavoisier's* views.

3. *Lymphatic System.*—Herophilus and Erasistratus had seen white vessels in the mesentery of some animals, and mistook them for arteries full of air. Galen, who never saw them, regarded the observation as chimerical, and taught that the veins of the mesentery absorbed the chyle from the intestines, and carried it to the liver, where it was transformed into blood; and his opinion was dominant until the middle of the 17th century. Eustachius had, in 1563, described the thoracic duct in the horse, without however suspecting its use. In 1622, *Gaspard Aselli*, dissecting a dog who had been killed shortly after feeding, observed several white filaments in the mesentery, which he mistook for nerves. Having accidentally pricked one, he was astonished to see a creamy fluid flow out; and a repetition of the examination in other dogs enabled him to establish the existence of the *lacteals*, as also of their valves. He believed they all went to the pancreas, and thence to the liver, which was then the acknowledged organ of sanguification. In 1647, *Pecquet*, while yet a student, discovered the *thoracic duct*. He also shewed that the lacteals did not go to the liver, but emptied themselves into this reservoir—thereby inflicting the final blow upon the doctrine which for ages had declared the liver the organ of hæmatosis. The investigation of the *lymphatic* glands and vessels henceforth occupied the attention of a great number of celebrated anatomists, as Vesling (who indeed discovered the thoracic duct about the same time as Pecquet), Bartholin, Ruysch, Rudbeck, the Hunters, Hewson, Cruickshank, and Mascagni, who published (1787) the first plates of the entire lymphatic system.

4. *The Nervous System.*—Hippocrates and his successors in the School of Cos possessed no precise idea of the functions of the nervous system, confounding, under the name of nerves, ligaments, tendons, and the nerves themselves; and it would seem that to Herophilus, the famous professor of the School of Alexandria, is due the earliest correct notions upon the subject. He describes three sorts of nerves; the first, which serves for the sensations and voluntary movements springing from the brain and spinal marrow; the second and third being destined to unite the bones together, and these to the muscles. Herophilus thus had not entirely thrown off the old error of confounding nerves and tendinous structure, and Galen was not exempt from similar views. He describes the brain and its membranes with accuracy, stating his belief that the cerebrum is the seat of the mind, and the origin of the nerves of sensation, while the cerebellum and spinal marrow give rise to those of voluntary motion. He maintains, however, that the extremities of the nerves are expanded into ligaments, membranes, and tendons; and places the sense of touch in the membranes covering the muscles, which have indeed, from their supposed origin in the nervous substance, received their name of *aponeuroses*. The anatomists of the 16th century described the distribution of the vessels and nerves with more accuracy; but most of the ancient errors on sensation and motion continued to prevail; and even in the 17th century Baglivi derived all vital movements from the heart and dura mater. The researches of Vieussens, Haller, Meckel, Vicq. d'Azyr, Soemmering, &c., during the last century, however, sufficiently demonstrated the true functions and structure of the organ. The opinions which attributed important functions to the dura mater, and the derivation of membranes from nervous ramifications, were proved to be destitute of foundation. It was shown that the medullary pulp of the nerves of the senses receiving the impressions transmitted them directly to the brain. The ancients had been more complex in their contrivances, and believed, as taught by Empedocles, that an elementary affinity prevailed between external objects and the organs of the senses —each organ possessing its element capable of attracting similar molecules of external bodies. The eye, for example, being of a resplendent nature, attracts the luminous molecules of bodies; the ear, of an airy nature, those which are sonorous, &c. Galen only added to the hypothesis the doctrine of spirits which were secreted by the brain, and transmitted to the organs of sense, visual spirits to the eye, where they entered into contact with the luminous particles of bodies, &c. Such were the ideas which prevailed even to the 17th century. At that period Kepler shewed the crystalline lens was not, as believed until then, the organ of vision, but an instrument for refracting the image of objects upon the retina; and Scheiner demonstrated this expansion to be the true organ of sight. The researches of Newton on light and colours much contributed to the establishment of a true theory of vision.

The *functions* of the nervous system have much occupied the attention of the moderns. Some have regarded the nerves as delicate tubes containing a subtle fluid, which, receiving the impressions of objects, transmits them to some central point of the encephalon. Others look upon the nervous fibrils as cords springing from a common centre. Bonnet revived the hypothesis of Hartley, which supposes every nerve to contain as many distinct fibres as it receives different sensations. Thomas Willis first assigned special functions to various portions of the brain, as common sense to the corpora striata, memory to the cortical substance, &c. Cabanis likened the functions of the organ to those of other viscera, it producing all the operations of the intellect, just as the stomach and intestines accomplish the digestion of food or the liver secretes bile. All these were mere hypotheses; but others founded their opinions upon observation, as Camper, in that which he estimates the mental development by the facial angle. Pinel, from his observations upon the insane, came to the conclusion, that the various faculties of the brain must have a special affection to a special portion of that organ; and it was at the end of this period that Gall commenced announcing the results of his prolonged investigations.

5. *Generative System.*—Galen described the male apparatus with accuracy, and recognized the analogy of the organs of the two sexes—the male organs being developed externally by the heat of the temperament, while those of the female were retained within by reason of her natural coldness. He called the ovaries the female testicles, and believed they secreted a fluid during copulation analogous to the semen of the male. Dissecting only animals, he believed the uterus was divided into two cavities. The male and female semen becoming mixed in the womb, the latter serves for the nourishment of the other, and for the production of one of the fœtal membranes. The male semen is almost at once converted into membranes, some of which afterwards become expanded and hardened, and modulated into the various parts of the fœtal frame. With Hippocrates, Galen believed the right testis furnished the material for male ova, the left for female, the female embryo always becoming developed in the left, and the male one in the right cavity of the uterus. These views remained entirely unchanged until the 16th century. Even then the chief anatomical errors of Galen only were rectified; and it required the successive investigations of Fabricius, Harvey, and De Graaf to bring our knowledge cf the true structure of these organs to its present state. As to the theories of generation, which may all be reduced to the two principal hypotheses of epigenesis and evolution, M. Renouard observes that, after the endless disputations it has led to, the question is as undecided as ever.

6. *The Vital or Organic Properties.*—Philosophers and physiologists of all periods have agreed in recognizing under the term essence, nature, soul, spirit, pneuma, &c., the existence of a primary intrinsic power presiding over the various functions of the body—as well as that of the physical and chemical properties in common with inanimate matter. Different observers, however, attached different degrees of importance to these.

"Some were especially attentive to the intrinsic force, studying with care its tendencies, and scrupulously following its indications: they were termed *Hippocratists.* Others, the *Humorists,* of whom Galen is the chief representative, had especial regard to the elementary qualities (heat, coldness, &c.) of the humours. Others, again, only took into account the physical qualities of the solids, and especially their porosity, and the power possessed by the various tissues of dilating or contracting. These were the *Methodists.* The *Empirics* disdained all physiological considerations, in which they were wrong, as in so complex a study as that of disease we should avail ourselves of all the light any branch of science affords. They might however have thus addressed other sectarians:— 'You are aware that the phenomena of the animal economy are the product of three description of forces, and yet you direct your attention exclusively to one of these, reckoning the others for little or nothing. But examine any function, the secretion of saliva for example—is it not evident that the vital, the physical, and the elementary or chemical forces, are here acting in unison? and who can say the exact part which each takes in the act? No one: and it is impossible to form an exact idea of the function as long as we separate by a mental analysis the forces which concur to its production. We must study it as it presents itself to our observation, synthetically and experimentally.'

"At the revival of science the old physiological systems were re-produced under different forms. The *Iatro-mathematicians,* versed in the calculation of physical forces, professed to explain the functions of the economy by the laws of mechanics. They saw only in the circulation, the secretions, nutrition, &c., the effects of the elasticity of tissues, the size of vessels, the friction of their contents, &c. The *Chemical Philosophers* gave exclusive consideration to the mixtures of the chemical elements, and spoke only of acid or alkaline humours, gases, salts, fermentations, &c. The *Hippocratists* regarded only the influence of the intrinsic force of the living body which they named *archæus,* vital principle, &c. From this it is evident, that physiologists recognised no difference between the properties of organized and inorganic matter; and, to explain certain acts of the economy, had recourse to the intervention of an immaterial or quasi-immaterial substance, as the soul, archæus, &c. They were ignorant of the *vital forces* properly so called."—*Tom.* II., p. 192.

Towards the middle of the 17th century, however, *Francis Glisson* admitted in the living solids a special force, with which all tissues are endowed in different degrees, and which he believed sufficed to explain all the vital phenomena. This he termed *Irritability.* His views were neglected and forgotten for more than 60 years, when they were revived by De Gorter. Irritability was however confounded with elasticity, until *Haller* (1747), by a series of most ingenious experiments, raised Glisson's hypothesis into an established fact; and clearly defined the differences between this vital contractility and mere contractility of tissue. Between 1757 and 1766 he published his unrivalled *Elementa Physiologiæ Corporis Humani.*

"Throughout the whole of this work he proceeds with his ordinary circumspection, advancing nothing that is not capable of support by well-ascertained facts, and according to hypothesis the smallest possible space. Rich in a multitude of original observations, and displaying a vast erudition, this work constituted an imperishable monument in the science of life. Dating from this epoch, physiology claims a separate existence from physics and chemistry, it having been demonstrated that life has its laws and special forces, which must be studied in an entirely peculiar manner."—*Tom.* II., p. 194.

The truths proclaimed by Haller keenly attracted the attention of the learned. *Fontana* was one of the most skilful of the defenders of the new doctrines, and *Bordeu* applied them to the explanation of the secretions, the variety of which he attributed to the specific sensibility of the different glands. *Fabre* first applied them to pathology in refutation of Boerhaave's mechanical theory of inflammation. "Haller had only demonstrated the existence of irritability in muscular fibre. His disciples admitted the property in other parts; but its existence in every tissue had yet to be proved by experiment and severe analysis. Its forms and degrees had yet to be exhibited—in a word, the theory of the vital properties required systematizing and extending to every function; and this task was undertaken with as much genius as boldness by the celebrated *Bichat.*" In the mean time, Hunter's beautiful experiments had fully established the vital properties of the blood; and various other writers had contributed to the progress of anatomy and physiology during this period, especially Winslow, Albinus, the Monros, Douglas, Vicq d'Azyr, &c. &c.

### 2. *Hygiene.*

During the period we are now occupied with, the subject of Hygiene much engaged the attention of physicians, philosophers, and statesmen. Its consideration is divided by M. Renouard into two sections, the one treating of *Public Hygiene,* the other of *Personal Hygiene.* Under the first of these headings he passes briefly in review the gymnastic exercises of the Greeks, the architectural contrivances of the Romans for securing the salubrity of their towns, the leper hospitals of the Middle Ages, and the establishment of lazarettos in the 17th century; he adverts to the improvements in the health of prisoners, soldiers and sailors, which have resulted from the labours of so many philanthropists and physicians; and, lastly, gives some account of the discovery of vaccination by Jenner.

Among the works on *Personal Hygiene* which the period produced, the "De Medicinâ Staticâ Aphorismi" of *Sanctorius,* in which he details the elaborate series of experiments he performed upon the transpiration of the body, deserve especial mention from the celebrity they acquired.

"This publication was received as a revelation, a true code of hygienic laws. Its author was saluted as a second Hippocrates, and his sanitary maxims placed on the same level or above those of the 'old man of Cos.' His salary as professor at Padua was continued to him after he had quitted Padua, and a statue was erected to him at Venice, where he died in 1636.

"Yet, if we submit his work to a severe criticism, we find it faulty in several respects. His conclusions are too general and absolute; for, from experiments made upon one individual in one climate, he draws inferences to be applied to every person in all kinds of climates. Moreover, many indubitable sources of error were mixed up with his calculations. Thus, he makes no account of the pulmonary exhalation or absorption or of the cutaneous absorption. Lastly, many observers in different countries having repeated Sanctorius's experiments have obtained very variable results. Whence it appears that there is nothing so mobile as the cutaneous transpiration, and that the design of estimating its quantity is, as Bichat says, as vain as to pretend to specify the volumes of water which are hourly vaporized from a source whose energy is varied every instant. The only general conclusion we can draw from these experiments is, that in the state of health this excretion is ordinarily very abundant, that it diminishes in old age, and that in all cases it merits the attention of the physician as well as of the physiologist. If, therefore, the 'Aphorisms' do not justify the enthusiasm their appearance excited, neither do they merit to be so utterly neglected as they are at present. Boerhaave, whose opinion should carry some weight, declared that no other medical work was so perfectly executed, and Lorry has added commentaries to it worthy of perusal at all periods."—*Tom.* II. p. 210.

The works of Cheyne, Fischer, Rammazini, Lorry, Juncker, Beddoes, Hallé, Tissot, Sinclair, &c., &c., were also among the most celebrated of those devoted to hygiene appearing during this period.

3. *Pathology.*

"Pathology during this period was viewed under very different aspects which we can only here summarily indicate, as we shall have to speak of them more at large when treating of the various theories. Some made the humours play the principal part in the generation of disease, conformedly to the modified Galenical doctrines or of the new chemical ideas; others only saw in every morbid disorder some error or disturbance of the regulating principle of the economy which they termed archæus, soul, nature, vital principle, &c.: others considered diseases as a dynamical derangement of the action of the solids; and, lastly, some rejected all consideration of causes and phenomena which did not fall within the cognizance of the senses, and decided to confine themselves to simple observation. From these different modes of viewing diseases very varied pathological classifications resulted, and in the end a much more profound and complete acquaintance with morbid conditions."—*Tom.* II. p. 213.

*Semeiology.*—Several physicians of this period made some one symptom the subject of special and abstract study. The variations in the amount of transpiration especially occupied the attention of Sanctorius, and several other observers paid the same minute attention to the conditions of the *pulse*. Among these, *Bordeu* endeavoured to attach all the shades of health and disease to certain determinate varieties of it, creating in this way endless subtle distinctions of doubtful reality and of no practical value. At about the same period (1761), *Avenbrugger* published his work upon *Percussion,* but which met with little attention, until translated and commented upon by Corvisart in 1808.

*Pathological Anatomy*, from the commencement of the 17th century, made rapid strides under the guidance of Bartholin, Tulpius, Ruysch, &c.; and the now immense number of observations were collected, examined, and classified by *Bonet* in his *Sepulchretum,* published at Geneva in 1700, an imperfect and confused work, but which, however, furnished the idea of his Anatomico-pathological Letters to the immortal *Morgagni,* published in 1762.

"In fact, he only proposed to himself, in composing these Letters, to amend and in some sort refound this work of Bonet. Profiting by the riches which science had acquired in the interval, and especially by those which his master, Valsalva, had amassed, and joining to these an extensive erudition and a severe critical spirit, he established order and clearness where the author of the *Sepulchretum* had left confusion and darkness. He shewed himself original without having the pretension of being so, contrary to so many others who possess the pretension without the reality. He disguises nothing that he borrows, whether from the dead or the living; but what he borrowed from no one was the selection and judicious employment of his materials, and the wise and luminous discussion of facts. Persuaded that medical science can only progress by the light of observation, he scrupulously avoids losing himself amidst vague interpretations; so that the idea quoted from the Odyssey in his preface may not be applied to himself, 'He has told much untruth in relating what is seemingly true.'"—*Tom.* II. p. 221.

From the period of the publication of Morgagni's "*Epistolæ,*" in 1762, to the end of the 18th century, a great number of enquirers diligently occupied themselves in anatomico-pathological researches, as Walther, Sandifort, W. Hunter, Greding, Lieutaud, Portal, and, above all, *Xavier Bichat.*

"Uniting to a genius eminently adapted for generalizing, an admirable talent for observation and analysis, he shed not only on pathological anatomy but on entire pathology, a bright light whose rays guided the labours of the majority of his successors. The idea of decomposing the animal body into elementary tissues, which present in every part where they are found the same properties, and are liable to the same changes, has been the parent-idea which, for sixty years, has served as the basis of the researches of pathologists."—*Tom.* II. p. 222.

*Nosography.*—Although the Hippocratic writers acknowledged the division of diseases into epidemic, endemic and sporadic, and into acute and chronic, yet they did not always observe this very strictly; and it was only after the foundation of the Alexandrian School, and under the influence of the perapetetic philosophy, that any attempt at a systematic order was observed. Diseases were divided into external and internal, general and particular. The general diseases were supposed to affect the entire economy, without having any determinate seat, as fever, gout, syphilis, poisoning, &c., while the particular diseases were seated in one of the three great cavities of the body. To the classification of the Methodists we have already alluded. That introduced by *Plater* at the end of the 16th century, does not seem to have exercised much influence, as, long after, writers upon medicine, such as Morgagni, Sennertus, &c., still followed the ancient methods. Towards the end of the 17th century, naturalists had made great progress in the classification of the objects of their studies, and *Sauvages,* a young physician of Montpellier, after consulting with Boerhaave, published in 1732 his *Nosology,* constructed upon the principles recognised by the botanists. It excited little attention until some thirty years after, when its author, having revised and enlarged it, republished it as his *Methodic Nosology.* It then acquired the highest renown, in proof of which, it was the only work employed by Linnæus in his lectures at Upsal for twenty years. "Whatever discredit this description of works may have fallen into in our times, the Nosology of Sauvages will always worthily employ the attention of those who like to follow the progress and development of a science so difficult as pathology. Besides forming the first of a series of interesting productions, it offers the most complete collection of descriptions of disease and observations collected from all sources, hitherto published."

Our own Sydenham had long before expressed his desire that a history of diseases might be written, disengaged from all hypothesis, in which the only object should be the exactly tracing the sensible phenomena and the distinguishing morbid species by their constant and essential symptoms. But although Sauvages entertained the highest veneration for him, terming him the glory of England and the light of our art, he was far from strictly following his counsel, too often mingling with his histories of disease vague theories and hypotheses concerning their causes and nature. When his System, too, was more calmly examined, it was found that the multitude of genera and species he had created tended only to induce confusion in diagnosis, not being separated from each other by sufficiently well-defined and constant symptoms. Numerous physicians endeavoured to improve upon his plan, but it was not until the *Nosology* of *Cullen* (1785) appeared that any true progress was really made. "This much reduced the number of species, and distinguished them by better defined and less variable characters. It presented then a real improvement upon that of Sauvages, and obtained a universal vogue, which it maintained until the publication of the *Nosographie Philosophique of Philip Pinel* in 1798. This eclipsed all the preceding ones, and soon became classical throughout Europe. Six editions within twenty years testify to the confidence it has enjoyed." Pinel divided diseases into six classes, 21 orders and 84 genera. His classes are—1, Fevers; 2, Phlegmasiæ; 3, Active Hæmorrhages; 4, Neuroses; 5, Diseases of the Lymphatic and Dermoid Systems; 6, Undetermined, comprehending genera which are not sufficiently connected to link into general orders. In the last edition, the two latter classes have been reduced to one, under the title of Organic Lesions.

"Sprengel, writing at the commencement of the 19th century, speaks of Pinel and his Nosographie in the following terms: 'Faithful to nature and experience as Hippocrates, whom he constantly takes as his model, and formed by the study of the best works on medicine at all periods, Pinel has taken his place among the most skilful and learned physicians of our times. His work is a true masterpiece, as much from the excellent plan he adopts as from the depth and impartiality of his judgments.' "—*Tom.* II. p. 234.

Besides the Nosologies adverted to, others were published by Linnæus, Vogel, Macbride, Sager, Darwin and others: but these require no specific notice, as possessing no authority. "Sauvages and all the other nosologists, not excepting Pinel himself, although approving of Sydenham's advice, of recording only the constant phenomena and sensible characters of disease, all fall more or less, under some pretext or other, into the error of occupying themselves in searching for the occult causes of disease." The *Precis de Medicine* of *Lieutaud* is cited as a remarkable exception to this stricture.

"The mere avoidance of hypothesis, however, will not alone constitute a good nosology. Exact and detailed descriptions of each morbid species is a primary requisite. Too brief descriptions engender obscurity, which after error is the greatest defect in this kind of work. Classifications may vary without end, depending as they do upon the manner in which an author views his subject, and diseases, being very complex objects of study, may be viewed from many points of observation. But descriptions of each morbid species, when well made, preserve their value independently of all changes in classifications and systems; this has occurred to the histories of Hippocrates, of Aræteus, of Alexander of Tralles, and of all great observers."—*Tom.* II. p. 338.

### 4. *Therapeutics.*

The old and erroneous maxim of the remedial agency of the contraries was revived under new forms in this epoch, during which, also, various new maxims and modifications of old ones were broached: but, as these were all more or less connected with some physico-pathological system, their consideration may be delayed until these come under notice, and, in the mean time, M. Renouard briefly passes in review some of the ameliorations in the treatment of disease, as exhibited in the management of Small-pox, Syphilis, Intermittent Fevers, &c. which were achieved, and the new remedies, such as tartar-emetic, mercury, bark, ipecacuanha, belladonna, digitalis, &c. which were introduced.

"An important remark to be made is, that these great improvements were not made by virtue of, but in spite of dominant theories, these having, indeed, proved the greatest obstacles to their adoption. If mercurial medication was so long carried to the extent of a mischievous salivation, we must accuse the Galenic theory, according to which the virus of the disease circulated with the humours of the body, requiring evacuation for its expulsion. What was the reproach which the adversaries of Cinchona addressed to it? That it produced no sensible evacuation. As, in their opinion, founded on the authority of Hippocrates, Galen, and others, the proximate cause of fever could only be vitiated bile or phlegm, no medicine which expelled neither of these humours could radically cure it. The Stahlians made a still more specious objection. They declared that fever, being a natural and salutary effort of nature to rid herself of a hurtful matter, the cutting short a paroxysm was to act controversive to the vital principle, and in the end to do more harm than good. If vaccination itself encountered opponents is not this because the Arabs, who first described variola, propagated the opinion that the disease was innate in man, and concluded that the prevention of its spontaneous development was to oppose the endeavours of nature, and to imprison the enemy within our walls.

"From the avowal of all, therapeutics has owed the progress we have described to the purely experimental method, that is, to *empiricism:* not the ignorant and blind empiricism of charlatans, medicasters, and pharmacopoles, who, acquainted with the name of a disease, at once supply the drug required; but to enlightened and methodical empiricism, surrounded with all the positive indications derived from physiology, pathology, and the accessary sciences—to the empiricism of Sydenham, Morton, Torti, Van Swieten, Lieutaud, Stoll, Jenner, &c.—to that empiricism for which Sprengel so frequently apologises." (See Chap. 2 and 3 of his 16th Section especially).—*Tom.* II. p. 254.

### 5. *Surgery.*

Although the 16th century produced some names of note in Surgery, these were but exceptions, and this branch of medical science made little additional progress until towards the end of the 17th, from which period, through the whole of the 18th century, France, England, and Germany continued to produce names of imperishable celebrity. A great impulse was given to its study in France, by the foundation of the *Royal Academy of Surgery*, in 1731; but it is to our own John Hunter we are indebted for its elevation to the dignity of a science. Upon this point, we may quote the following passages from M. Malgaigne's Essay.

"If he suffered during his entire life from the absence of those preliminary studies, without which it is so difficult to impart to reasoning all its force and clearness of expression, we must at least do him the justice of testifying that he neglected no means of giving himself an education, and such a one as he deemed most suitable for the objects he had in view. Perhaps, too, strongly determined to consult only experience, he felt somewhat embarrassed in determining what part of our art he could most profitably apply his experience to. It is curious even to follow him in his efforts and hesitation. Anatomy had proved, in the hands of J. L. Petit, a powerful instrument of progress in surgery. John Hunter attached himself to its pursuit, consecrating to it ten years of his life; and, after having drawn from it some rich views, such as his theory of congenital hernia, perceived that he had mistaken his route and returned. What human anatomy could not furnish him with, perhaps he might find in the study of comparative anatomy. In this he engaged with renewed resolution, maintained a menagerie at his expense, stole hours from the night to expedite his dissections, and thus accumulated with his pen and pencil the anatomical history of three hundred and fifteen different species. But comparative anatomy not even serving his ends, a new ray of light broke in upon him. The subject is too often dumb, and he determined to interrogate the living. He instituted experiments upon animals, and thus created that fertile instrument of verification and progress, experimental surgery. Then, as he knew better than any other what differences separate man from the inferior beings, he recognized the necessity of controlling his experiments by direct observation on the living and dead man, and founded *Surgical Pathological Anatomy*. He resolutely approaches the fundamental question, from which J. L. Petit himself had shrunk, and enquires where are the bases and principles of surgery. Fear not that he will lose his time in merely examining the hypothetical theories of Boerhaave or of any one else. He does not even deign to mention them, and they are for him as if they had never existed. It is in the region of pure observation that he professes to delve, and it is there he hopes to lay foundations which shall prove indestructible.

"A curious comparison of dates may here be mentioned. The Royal Academy of Surgery in 1774 published its last volume of Memoirs, and in 1775 Hunter commenced his celebrated course of lectures on the Principles of Surgery. Thus, during the whole period that the school of Petit was declining, the English surgeon was occupied in preparing the way for his own; and scarcely had the one lapsed into silence, before the other raised its voice and seized the noble inheritance. A memorable epoch, well worthy of celebration! Surgery, as the Middle Ages had left it, was little else than a handicraft: A Paré and J. L. Petit made an art of it; and John Hunter constituted that art into a science.

"It is to Hunter, in fact, that we owe those general principles, which, regulating all portions of the art, and connecting them to each other, have made of it a magnificent whole, and which furnish, at the same time, so much strength to its doctrines and safety to its practice. It is Hunter who has made the surgeon, in the beautiful language of Bacon, the interpreter and minister of Nature. It is he who has revealed to us the procedures she follows in the cure of most surgical affections, and has taught us how to direct her operations. The union of simple wounds—the nature of suppuration in the more complicated ones—the various phases of cicatrization—the varieties of inflammation, (that fortunate or redoubtable phenomenon according to whether we maintain it within due bounds or abandon it to its violence)—these are some of the subjects which Hunter has treated with an unrivalled superiority. All his views in these respects have so completely passed into the common domain, that the majority of surgeons put them into practice, in the idea that they have at all times been recognized, little dreaming of their recent origin. But take away from our classical treatises all that can legitimately be referred to Hunter, and you will see what an immense breach you have made, and how vast is the position he has created for himself in surgery."—*Bulletin*, p. 186.

M. Renouard details at considerable length the histories of the various surgical operations; but our limits quite preclude our following him.

### 6. *Obstetrics.*

The obstetrical division of medical science long continued in arrear of the other branches, being for the most part in the hands of ignorant women. One of these female practitioners, however, more enlightened than her *consœurs,* set an example, which has been followed by some illustrious members of the sisterhood in our own times, of publishing, at the beginning of the 17th century, an account of her experience; but it was not until 1666, when the first edition of Mauriceau's work appeared, that the art of the accoucheur was placed upon any firm or rational basis. Many of his contemporaries likewise published upon this subject; "while the labours of the surgeons and physicians of the 18th century have raised several portions of the art to a degree of perfection well nigh approaching that of the exact sciences."

The midwifery forceps were invented in 1721 by *John Palfyn;* for, although the Chamberlayne family had long before employed an instrument of this kind, they disgraced themselves by refusing to give it publicity, whereas Palfyn hastened to announce to the world the instrument he had contrived, and which was afterwards advantageously modified by Smellie and Levret in England and France respectively.

7. *Clinical Medicine.*

*Oral Clinical Instruction,* after having prevailed in the Asclepiadean families until the period of the School of Alexandria, fell into disuse, only to be revived at an epoch not very remote from our own, viz. in 1578, when it is related that the professors, Bottoni and Oddo, taught clinically at Padua. However the practice seems to have been interrupted, and not to have been formally re-introduced, until Otto de Hewin lectured at the bedside at Leyden, at the beginning of the 17th century. Leboe, commonly termed Sylvius, followed his example, and his lectures acquired an immense reputation from 1658 to 1672. Again the practice fell into disuse, when it was revived at Leyden by the illustrious *Boerhaave* (1714), whose talents and celebrity attracted auditors from every part of Europe.

"His renown, which was already great, for he had published his *Institutions* and *Aphorisms,* became immense. He was consulted from the most distant countries, and was in correspondence with several sovereigns, and the Pope himself, although a Protestant. In searching for the real titles which recommend this illustrious man to the admiration of posterity, we find them thus clearly laid down by M. Dezeimeris, in the Dictionnaire Historique de Medicine.

"'Boerhaave exercised, during his life-time and long afterwards, an immense influence upon medicine. Inferior in genius to his cotemporaries, Stahl and Hoffman, he enjoyed a much more extended reputation, and his doctrines long prevailed over those of his rivals. He owed this to the success of his mode of teaching, and the brilliant qualities of his mind. Gifted with rare activity and facility, he acquired the most varied and extended knowledge. Upon this he constructed a system connected together in all its parts with infinite art. Developed in his lectures and in his works with method, clearness, and precision, and set off with a rare grace of eloquence, we can believe it secured all suffrages. This system, which may be considered as a true eclectism, was formed of some ideas taken from Themison and the ancient methodists, those of the chemiater Sylvius, and especially of those mechanical and iatro-mathematical theories, towards which his taste and mathematical studies naturally inclined him. These last predominated, and this is why Boerhaave is justly classed among the mechanical physicians. It is to be regretted that, with such happy powers of observation, he allowed himself to be estranged from even his own principles by the spirit of system and hypothesis. He commenced by preaching the method of Hippocrates with enthusiasm, and finished by following the brilliant, but uncertain example of Galen.' " *Tom.* II., p. 311.

After the death of Boerhaave, the Leyden school rapidly declined in reputation, which was transferred to Edinburgh, and especially to Vienna, where Van Swieten, De Haen, Stoll, and Frank taught in brilliant succession.

The number of *written clinical observations* continued to encrease during the period, which is characterized by attempts at a more accurate classification of already acquired facts, and an especial study of the influence of climate, seasons, regimen, and epidemic constitutions. Towards the end of the 16th century, the Hippocratic methods of study beginning to prevail over the Galenic, these important subjects occupied much attention; the work of *Baillou* upon the *Epidemic Constitutions of Paris* during the years 1570-80, representing the period of almost insensible transition from the Galenism of Fernel to the Hippocratism of Sydenham and Stoll.

*Sydenham,* who flourished during the better half of the 17th century, has justly obtained the title of the "English Hippocrates," both on account of his medical doctrines, and his profound study of the Epidemic Constitutions. The friend and cotemporary of Locke, he first taught physicians to recur to the simple observation of morbid phenomena after the example of Hippocrates. So great, indeed, was his dislike to mere hypothesis, that Sprengel has not hesitated to place him among the votaries of Empiricism; but he too often departs from his own wise maxims, by adverting to the essential causes of fevers and other disease to render this allowable. Much as he admires the admirable patience and untiring zeal with which Sydenham observed the influence of the Epidemic Constitutions, during so many years, M. Renouard is disposed to regard the theory he broached concerning their stationary character, supported though it is by the subsequent opinions of Stoll and Pinel, as fanciful, and calculated to destroy all stability in therapeutics. This and other of the opinions and labours of our countryman we could have wished to have examined at some length, both on account of their intrinsic importance, and because we believe they are insufficiently appreciated by the practitioners of our times. We are therefore much pleased to find that Dr. Milroy is publishing a series of admirable papers in the pages of a cotemporary,* with the laudable intention of presenting a complete and faithful summary of the writings of this great observer.

### 8. *Theories and Systems.*

The rapid progress of the various branches of natural science had already much shaken the authority of the Scholastic Philosophy, when, at short intervals from each other, *Bacon* and *Descartes* appeared, who, however much they differed from each other in the characteristics of their genius, or the modes of their reasoning, agreed in demanding as the primary step the entire liberation of the mind from its trammels. The great innovation introduced by *Bacon* was the reasoning from individual ideas to general axioms, thus reversing the procedure of Aristotle. The *Inductive System,*

---

* Vide Lancet, for Aug. 15th and Nov. 14th, 1846, and for Jan. 16th, 1847.

in his hands, was however disfigured by vague descriptions and departures from the lessons of experience, and met with little attention until it was completed and popularized by *Locke,* who, with lucid sagacity, demonstrated that which Bacon had only affirmed. All the leading philosophers of England and France now adopted it, and no one contributed more to its greater simplification and general reception than *Condilliac.* M. Renouard contrasts at some length the Inductive with the Deductive system of Descartes, Leibnitz, and Kant; but we need not pursue the parallel, as it is now generally acknowledged that, however appropriate the latter may be for conducting the investigations of the metaphysician, the moralist, and the mathematician, it is to the former we can alone look to for safe guidance in the pursuit of natural science.

### *The Origin of Animism and Chemical Medicine.*

No one contributed more to the discredit of the ancient philosophy and the introduction of a taste for novelties than did *John Baptist Van Helmont* (1577—1644). Thoroughly versed in a knowledge of the ancient writers, attached to the mysticism of Thomas a Kempis and other divines, an able and eloquent disputant, a friend to independent observation, a discoverer in chemistry, and believer in alchemy, his writings constitute a transition period between the ravings of Paracelsus and the more learned theories of a later epoch. Yet, a keen exposer of the verbiage, the inconsistencies, and the visionary theories of Galen and Aristotle, he expounds in his turn a system so baseless and so confused, that M. Renouard, with all his patience, renounces the attempt of giving any connected view of it. According to it, the animal economy is influenced by three motor powers, consisting of certain *ferments,* the *archæus,* or great governing principle, and a third, which he terms *blas,* regulating the natural and voluntary movements. To the Stomach and Spleen, under the quaint title of the *Duumvirate,* he accords an omnipotence over the rest of the œconomy, the *archæus,* or sentient soul, always residing in one of these viscera, and especially at the pylorus. There are no less than six stages of *digestion* described, operated, through the agency of various ferments, in the stomach, the duodenum, the mesentery, the heart, and the brain. The primary seat of all *disease* is the lining membrane of the stomach, the abode of the *archæus* and the various symptoms result from the efforts made by this principle to rid itself of the morbid conditions which injurious agencies may have induced. In *therapeutics,* substances only which are agreeable to this *archæus* must be prescribed. Bleeding was proscribed and purgatives used sparingly, opium, wine, and the new mineral preparations being the favourite remedies, not neglecting magical words, charms, and amulets.

"Van Helmont founded no sect; but several sects borrowed from his ideas. The chemical school owes to him the idea of ferments, and from him the animists and vitalists derived that of the vital principle, modelled upon his *archæus.* The miracle-mongers, magnetisers, &c., place him among the adepts, and never did the partisans of the scholastic philosophy meet with a ruder adversary. 'At an epoch,' says M. Littré, 'when the superstitious beliefs of the Middle Ages were still adhered to, and when the powers of Nature, timidly interrogated, seemed always to present themselves under a mysterious or supernatural form, we should not feel surprised at the mystical spirit of Van Helmont, at his ecstacies when he saw his soul, or at his dreams, during which the solutions of the most difficult problems were revealed to him. Nor is it astonishing that he often substituted hypothesis for hypothesis, error for error. The observers of that period were, in regard to many questions now well understood by us, in the same condition we find ourselves in with respect to other difficulties insoluble by our modes of investigation. What theory have we to offer in explanation of the cure of ague by bark, the origin of variola, or the destruction of its germ by vaccination? Who of us has not made his vain efforts to penetrate obscurity, and plunge beyond the horizon? Well! Let us then cast a glance back at that past which was then future, on our lights which were then darkness, and we can picture to ourselves the false glare and gropings of our predecessors—all the more ready to lose themselves, as unpossessed of that compass we have—the method of observation—inasmuch as, in the absence of facts, they could scarce abstain from hypotheses."—*Tom.* II. p. 378.

*Iatro*-Chemistry.—The Chemist-Physicians.*

*Francis Leboe,* surnamed *Sylvius* (1614-1672), the first Clinical Professor at Leyden, and a great cultivator of anatomy and chemistry, first employed the laws of this last science for the exclusive explanation of the animal economy. Borrowing the idea of the agency of *ferments* from Van Helmont, he does not employ the intervention of his *archæus* or governing principle. The saliva, bile, and pancreatic fluid, from the active parts they play in the economy, are termed the *triumvirate,* and through their agency the various fermentary processes are gone through. Professing to found his views entirely upon observation, he is continually assuming what is not proved, and explaining what is not understood. Disease, according to his views, consists in a vitiated or acrimonious condition of these various fluids, and is best treated by purgatives, narcotics, and the abundant use of volatile alkalis for the correction of morbid acidity.

*Thomas Willis,* (1621-1625), our countryman, and the author of so excellent a work upon the brain, basing his views upon the same chemical foundation as the Leyden professor, even surpassed him in gratuitous hypotheses. From the igneous analysis of bodies he concluded there were five elements, spirit, sulphur, salts, earth and water, and endowed these with qualities as fanciful as the ancients attributed to theirs. The various organic apparatus of the economy, by virtue of special ferments with which they are endowed, are enabled to produce the requisite proportions of these different elements; and it is through the faulty operation of such fermentations that disease is generated; so that the physician has little else to do than watch such operation and remove obstacles from its due performance. Remedies act upon the spirits or the humours, exciting or modifying their fermentory motions in a thousand manners, thus producing various effects upon the system, and secondarily modifying the condition of the solids.

*Iatro-Mechanics.—The Mechanical Physicians.*

As the progress of chemistry gave rise to the attempt at explaining the functions of organized beings by the same laws which regulate the elementary combinations of inanimate matter, so that of mechanical and mathematical science suggested the application of calculation to the elucidation

* Ιατρος, *Medicus.*

of the same phenomena. *Sanctorius,* by his experiments upon transpiration, led the way in this new description of research; but *Alphonso Borelli* (1608-1679) was the true founder of the Iatro-mechanical Sect. He published several essays bearing upon the subject; but his great work *De Motu Animalium,* did not appear until the year after his death.

"The fruit of patience and genius, this work created a new branch of medicine. Until then, very vague and erroneous ideas prevailed as to the amount of force expended by animals during their various movements, and the manner in which it was applied. Setting out from the principle that Nature attains her ends by the most simple means and the most direct road, it had always been believed that man and animals were so constituted as to be able to execute the greatest movements and bear the heaviest burthens whilst employing the least possible power. Borelli overcame this prejudice by reasoning founded upon anatomical research and the laws of statics. Comparing the bones brought into play by the muscles to cords set in motion by levers, he proved that man expends an enormous amount of force during his movements. * * * * * * However much some of his calculations may be wanting in exactitude, and accepting them only approximatively, it is still demonstrated that man develops, during his movements, an incomparably greater amount of muscular energy than the obstacle he has to overcome—a truth which was far from being suspected prior to the time of Borelli. His book contains also a prodigious quantity of observations as minute as new, upon the various modes of progression and the postures of animals. To cite one example of a thousand, he furnished a very ingenious explanation of the manner in which a bird supports itself upon one foot while sleeping, and that upon a branch of a tree which every breath of wind puts into motion."—*Tom.* II. p. 393.

Really possessed of as little foundation as iatro-chemistry, Borelli's mechanical explanations of all the healthy and diseased phenomena of the economy, seemingly supported by anatomical facts, minute hydraulic calculations, and the recent microscopic observations, obtained, by their apparent mathematical exactitude and simplicity, the suffrages of numerous enlightened observers. No such precision of ideas had been attempted since the porous tissues and various shaped atoms of the old Methodists. Bellini, Baglivi, Sauvages, Senac, Boerhaave, Bernovilli, Pitcairn, Keill, Freind, and Mead, were among those who accepted them with more or less modification. Of these, M. Renouard confines his attention to Baglivi and Boerhaave.

*George Baglivi* (1668-1706), surnamed the Roman Hippocrates, endeavoured to extend the application of Borelli's ideas to pathology and therapeutics. Believing that the prevailing sects paid too exclusive attention to the condition of the *humours,* he set himself the task of proving that in all conditions of the economy, whether of health or disease, the *solids* are of predominant importance. Like the Methodists under Themison, he admits of only two classes of affections of the primary fibril, viz. too great tension or constriction, and too great softness or relaxation—the *strictum* and *laxum.* But Baglivi's theories are not his only claim to our notice; for he produced an admirable work on practical medicine, in which he insists upon our taking observation as our guide, sacrificing theory to experience. We no longer see merely the great opponent of the old humoral pathology and parent of modern *solidism;* but the enlightened practitioner, admitting that, in chronic disease, there may be a cocochymia or vitiation of the humours.

*Hermann Boerhaave* (1668-1738), endowed with a vast and subtle intellect, and profoundly versed in the writings of the ancients and the labours of the moderns, conceived the idea of uniting in one body of doctrine the various theories of medicine. Like Galen and Fernel, he was an Eclectic, but, inasmuch as mechanical explanations predominate in his writings, he has been classed with the Iatro-mathematicians, just as they were with the Dogmatists. He published his *Institutiones* in 1708. Their physiology consists of a most skilful amalgamation of anatomical, physical and chemical ideas. Unlike Baglivi, however, he did not, in the practice of his art, abandon his speculations for observation, and never seems for an instant to have doubted their exactitude.

After the death of this celebrated man, the iatro-mechanical doctrines rapidly fell into obscurity, from which historians of physiology have only revived them.

*Animism and Vitalism.*

At the very time when the celebrated Leyden professor was spreading far and wide his mechanico-chemical theories, the newly-founded school of Halle was producing observers whose systems were speedily destined to overthrow these. *George Ernest Stahl* (1660-1734), conferred a vast benefit upon the study of the science of life, by recalling the attention of its votaries to the contemplation of the effects of the vital powers upon the economy in health and disease. The chemists had presented their ferments as the essential phenomenon of life, which the mathematicians placed in the contraction of the primitive fibre; but Stahl makes it consist in the preservation of the integrity and due mixture of the humours of the body through the immediate agency of the *anima* or immaterial soul. This agency he endeavours to prove by two arguments: first, that the body has been only created as the mere instrument through which the soul might operate; and secondly, that motion, by which alone life is maintained and its actions carried on, is a spiritual, not a material act. Unstable as is this hypothesis, it has the merit of greater simplicity than some of its predecessors, which its inventor ridicules with the bitterest irony. It is, in fact, but a modification of the *archæus* of Von Helmont, and did good by recalling attention to the study of the vital, as distinguished from the mere mechanical, phenomena then in vogue. Every pathological condition, according to Stahl, resulted from the re-action of the *anima* against the morbigenous principle; and as the symptoms of disease but represented the regular succession of a series of vital movements designedly excited by a reasoning agent, the office of the physician became reduced to that of a mere spectator of the sufferings of the patient, since active interference on his part might only derange some of the combinations of this supreme regulator of the economy. The iatro-chemists and mechanicians had too much lost sight of the great power which nature possesses of rectifying the derangements of the economy, just as the Stahlians grossly exaggerated it.

Out of Germany the doctrine of Animism made but few converts, and in France that of *Vitalism*, as advocated by Barthez, of Montpellier, was far more generally received. This recognised the agency of the *vital principle*, much more resembling the *archæus* of Von Helmont, than did the *anima* of Stahl, distinct from the body or the immaterial soul, and yet endowed with feeling and perception.

*Organic Dynamism, (Vital Power resident in the various Organs.)*

Another class of physiologists believed the *vital forces* to be inherent in the respective organs, and occupied themselves in studying the laws of their operation. *Hoffman* (1660-1742), who first set the example of this simpler mode of viewing the economy, regarded disease as resulting from a perverted condition of the vital movements, too great contractility inducing spasm, and too feeble relaxation. These movements of the organic solids were, however, considered by Hoffman but as effects of the elasticity of structure; and *Cullen* (1712-90), was the first who applied the results of the researches of Haller upon the contractility and irritability of tissues to the construction of a medical theory, assuming irritability as a primary fact, the origin of which it were futile to search for. Hoffman regarded an anormal afflux or reflux of blood as the primary instrument of tension or relaxation, Cullen sought the point of departure in the nervous fibrils. Both, however, were admirable practitioners, and, notwithstanding their partiality to solidism, admitted of medicines calculated to act upon the humours, and frequently exhibited the therapeutical indications derived from the observation of the apparent phenomena with remarkable clearness. *John Brown* (1735-88) attempted to build upon a portion of Cullen's theory a most fallacious and dangerous system. He made health consist in the maintenance of a normal amount of excitability, disease being of a sthenic or asthenic nature, accordingly as this is in excess or defect; and, as he considered the vast majority of affections to be of a hyposthenic character, the exhibition of active stimuli constituted his principal therapeutical agent.

*Revival of Rational Empiricism.*

The partisans of the ancient medical sects, however much they differed among themselves, united in opposing the experimental methods of the Empirics, which so tended to sap the very foundation of hypothetical reasonings. The consequence was that universal odium and neglect befel these enquirers, who were really in advance of their age, and it has not been until recent times that their principles have been avowedly adopted. The progress of the Inductive philosophy should seem to be highly favourable to their reception; but, through prejudice and the abstruse nature of medical science, and the difficulty of discerning in it the reality of experimental deduction, the bearing of medical enquirers during the Reformatory Period towards Empiricism was most uncertain and contradictory.

"It would be easy to exhibit such contradictions in the writings of Torti, Sydenham, Stahl, Morgagni, Sauvages, Cullen, Barthez, Pinel, Frank and others, in all of which we find the maxims of experimental philosophy adopted and proclaimed, and yet the name of empiricism discarded and denounced."

We regret our inability to follow M. Renouard through his interesting exposition of the recent triumphant progress (especially in Britain) of the principles of Rational Empiricism. Indeed, notwithstanding the length of this article, we have been compelled to pass over the last chapters of the work in a very hurried manner; and, entirely analytical as our notice has been, we can find no room for any comments or criticisms it suggests. These must be reserved until the publication of the Supplement, in which M. Renouard proposes to sketch the doctrines of the 19th century. In the mean time we may express our cordial approbation of the manner in which he has executed his present elaborate performance.

